# War can harm intimacy: consequences for refugees who escaped Syria

**DOI:** 10.7189/jogh.09.020407

**Published:** 2019-12

**Authors:** Niveen Rizkalla, Steven P Segal

**Affiliations:** Mack Center on Mental Health & Social Conflict, School of Social Welfare, University of California Berkeley, Berkeley, California, USA

## Abstract

**Background:**

Syrians seeking refuge have been exposed to atrocities and trauma beyond comprehension. This study examines how personal, interpersonal, displacement and war-related factors have impacted married refugees’ intimate lives.

**Methods:**

Data included 158 married Syrian refugee individuals who live in the host communities of Jordan. Refugees reported on their personal, interpersonal, current-displacement and past-war related experiences. Traumatic impacts were assessed using the Harvard Trauma Questionnaire (HTQ), K6 screening scale for serious mental illness (SMI), The War Events Questionnaire (WEQ), and Personal Assessment of Intimacy in Relationships (PAIR). Stepwise multiple regressions were used to determine the factors associated with refugees’ intimacy-total score and its six dimensions.

**Results:**

Most refugees (94.2%) experienced war events, and 34% screened positive on the PTSD-HTQ scale. Overall intimacy scores were low, scoring M (±standard deviation) = 2.4 (±1.1) of a possible five on average. Intimacy scores were lower for refugees who screened positive on the PTSD-HTQ (M = 1.95 ± 65) compared to the ones screening negative, respectively (M = 2.23 ± 66). Furthermore, the higher the PTSD symptoms reported, the lower the couples’ intimacy. PTSD and forced marriage were the strongest factors to predict decreased total-intimacy scores (β = -0.23, *P* = 0.002; β = -0.32, *P* < 0.001), and decreased scores on four dimensions of intimacy (emotional, sexual, intellectual and recreational). Whereas gender was the second strongest factor associated with decreased total-intimacy scores (β = -0.29, *P* < 0.001), and decreased scores on three dimensions of intimacy (emotional, social and anger), meaning that women reported suffering more than men from deteriorated intimacy in their marital relationships. Other displacement and war-related factors associated with intimacy were: decreased sexual intimacy associated with having been raped; increased intellectual intimacy associated with escaped from Syria with one’s spouse; decreased recreational intimacy associated with the number of family members lived with; decreased sexual, emotional and total-intimacy scores associated with number of children; and years of education as a seemingly personal protective factor associated with increased intellectual and recreational intimacy.

**Conclusions:**

Addressing Syrian refugees’ intimacy issues in interventions is essential, as well as raising the awareness of stakeholders and community leaders to the negative impacts of PTSD, forced marriage, rape, and displacement difficulties endured by the already challenged and distressed married refugees.

The ongoing Syrian conflict has taken a toll. This study examines how personal, interpersonal, displacement and war-related factors have impacted married refugee individuals' intimate lives. There are diverse definitions to intimacy, but the common component to all is the sense of closeness and connection that develops through communication in couples’ interactions [1]. A profound research literature has been written on the consequences of conflict related posttraumatic stress disorder (PTSD) for combatants and the secondary traumatization of their family, especially their intimate partners [2,3]. However, much less knowledge exists pertaining the impact of war on the family system in general, and on couples’ intimate relationships in particular [3-6].

Theoretically and clinically, there are multiple dimensions of intimacy [7]. However, only five (emotional, social, intellectual, sexual, and recreational) have been empirically validated [8]. Emotional intimacy is defined as experiencing closeness, sharing feelings with one’s partner and feeling supported; social intimacy is related to couple’s experience of having common friends and similarities in social networks; intellectual intimacy is the experience of sharing ideas about life and work, and respecting the other’s ideas even when disagreeing with them; sexual intimacy defined as the experience of sharing general affection, which includes physical and sexual activities; and recreational intimacy is the shared interests in hobbies, mutual sport events, and recreational activities [7,8].

Studies show that exposure to war, as a combatant who had developed PTSD, is associated with impaired couples’ relationships [9,10]. Combatants with PTSD who start new intimate relationships or returned home to their former partners after experiencing traumatic events (post-deployment), not only brought the PTSD symptoms to the dyad, but also physical impairment, high rates of alcohol and/or drug abuse, and psychological and physical aggression, or intimate partner violence [10,11]. Researchers therefore suggest that individual trauma and displacement stresses affect refugees at the individual and familial levels [12].

Issues causing conflict between refugee couples in the host countries have been identified as finances management, lack of family and social support systems, lack of daily communication, acculturation stress, and limited knowledge of official procedures [13]. Others suggested that loss of legal status, economic hardship, working hours, lack of family support, and changes in gender roles and responsibilities have increased marital distress and separation [14], increased poverty and property loss [15,16], as well as aggression and intimate partner violence against women [17]. A study on post conflict Uganda reported that poverty, limited educational opportunities, and weakened family structure, affected marriage and youth sexual practices [18]. Another study on refugee women who lived in three refugee camps in South Sudan, Kenya, and Iraq identified factors such as women’s separation from family, rapid remarriages, and forced marriages exacerbate women’s risk of experiencing intimate partner violence [17]. Furthermore, a study of the aftermath of Rwanda’s genocide found that couples experienced structural changes in their partnership that may have led to conflicts and separation [15]. A study related to Syrian and Palestinian-Syrian refugee women in Lebanon found that they suffered from intimate partner violence, early marriage, and survival sex due to the Syrian conflict [19].

Other researchers report positive effects of displacement on marital relationships, such as increased self-reliance and shared decision making of couples, in the absence of family support, which in turn may enhance couples’ intimacy, affection and communication [14,20]. Perhaps the distress among war-affected-populations is due not only to traumatic exposure, but also to the continuous impact of displacement challenges [21].

Studies debate over gender differences, advocating that men and women vary in the risk of trauma exposure, the traumatic events experienced, and PTSD prevalence [15,22]. Women are more likely than men to develop PTSD after experiencing traumatic events, and their PTSD prevalence is greater than men [23]. Studies suggest that women are more vulnerable to experience sexual violence, especially in conflict zones [15], while men who experience sexual violence are at higher risk of developing PTSD [24]. Still, some studies didn’t find post war-PTSD differences among genders [15,25]. A study on internally displaced Syrians (IPDs) and Syrian refugees in the Netherlands found no significant difference between genders in PTSD. However, women had more suicidal ideations, while men demonstrated more suicidal attempts. Men reported experiencing more collective identity traumas and war events, more decrease in memory and loss of functioning, compared to women [25]. Researchers argue that such differences are mainly dictated by gender social construction, rather than actual differences [22].

Given the limited studies on the impact of conflict and its precursors on intimacy in refugee marital relationships, we hypothesized that: (1) Personal, interpersonal, displacement and war-related factors will have an overall negative impact on refugee couples’ intimacy. (2) These factors may also affect different dimensions of couples’ intimacy in both positive and negative ways. (3) Differences among refugee men and women will sharpen disparities among genders, which might be exacerbated by refugee displacement

## METHODS

### Participants and procedure

Interviews were conducted (N = 158) between March and August 2014 in Jordan (Amman (45.6%), Al-Zarqa (23.4%), Mafraq (13.9%), Ar-Ramtha (8.9%), Irbid (6.3%), and Hiteen (1.9%)). Inclusion criteria were: (1) Being a Syrian refugee living in an urban area in Jordan. (2) Being married (sincere answers related to intimacy in the Arab society would have posed a challenge if offered to non-married individuals). (3) 19+ years of age. (4) Agreement to participate in the study.

Interviewees were recruited at humanitarian non-governmental organizations (NGOs) in Jordan. Refugee-participants completed interviews during the organizations’ working hours. If participants could not complete the interviews during scheduled hours, the interviews were held in the evenings at public places (eg, restaurants or coffee shops) or at their homes. Other participants were recruited with the help of Syrians who operated as volunteers at the organizations and assisted in reaching out to the refugee population in the community. Refugees who refused to participate communicated their refusal to organization staff or volunteers, thus, the refusal rate is unknown to the researchers.

This study was approved by the Committee for the Protection of Human Subjects at the University of California, Berkeley. Participation was anonymous, voluntary, and without incentives. Syrian refugees only provided verbal consent (written consent was not required), to enable a safe and secure space for participation. The interview schedule started with demographics and experiences of the war and symptomology, and ended in the questionnaire regarding intimacy, allowing some time to break the ice between the researches and participants to provide as much as possible accurate responses to the sensitive topic of their relationships. The survey including original instruments were translated into Arabic by two independent professional translators and back-translated to English for both accuracy and cultural sensitivity. Five humanitarian organization staffers in Jordan assisted in further adjusting the scales to the Syrian refugee context. Additional edits were offered by a linguistics expert to the final version. Though the first author was responsible for conducting the majority of interviews, she recruited research assistants with mental health backgrounds and trained them on interviewing refugee traumatized populations in sensitive circumstances; she also referred participants to the adequate treating organizations if self-harm risks were exposed.

### Measures

Data collection included general information on refugees’ demographic and psychosocial circumstances: Age, gender, years of marriage, age at marriage, number of children, years of education, origin, circumstances of arrival to Jordan, housing/living conditions, employment, economic status and others.

The Personal Assessment of Intimacy in Relationships (PAIR) scale [8] was used to measure intimacy perceptions. The PAIR employed herein contains 34 Likert-type items, rated from 0 (strongly disagree) to 4 (strongly agree). Thirty items measured the five dimensions of intimacy (emotional, social, intellectual, sexual and recreational) and four items related to coping with anger in an intimate relationship [26]. The PAIR was originally translated to Arabic and validated with Palestinian couples [27]. The anger-related items were utilized in a different study in the Middle East [28]. The current sample’s total intimacy α = 0.90 (34 items). Its subscale alphas are: Emotional intimacy α = 0.88 (6 items); social intimacy α = 0.23 (6 items); sexual intimacy α = 0.80 (6 items); intellectual intimacy α = 0.81 (6 items); recreational intimacy α = 0.71 (6 items); and anger in an intimate relationship α = 0.44 (4 items).

The War Events Questionnaire (WEQ) [29] consists of two parts. This study only used the first part that assessed the occurrence of specific war events, their severity, and whether exposure happened in witnessing the event, experiencing it in person, or if it happened to a person close to the refugee. Participants were asked to answer the yes/no question: “Have you had any exposure to the experiences of war?” A “yes” response was followed by a request to elaborate on the experience. The Arabic version of this scale was originally developed and validated to examine Lebanese civilians’ levels of exposure to war [29].

The Harvard Trauma Questionnaire (HTQ) [30] is a 46 item Likert-type measure that assesses PTSD symptoms associated with traumatic experiences. We used the first 16 trauma symptom items to assess PTSD severity (1 = not at all to 4 = extremely), with higher scores indicating ascending severity. The current study utilized the Arabic version of the HTQ that was previously used with Iraqi refugees [31], with minor changes made to adjust it to the context of the Syrian crisis. The current sample’s Cronbach’s α = 0.89.

The K6 Arabic version, a screen for serious mental illness (SMI) [32], has been validated for use in Lebanon [33]. K6 symptom responses are standardized via a five-point Likert scale ranging from 1 (all the time) to 5 (none of the time) with higher scores indicating the absence of symptoms [32]. Scores for this six-item screen range from 6 to 30; a positive screen for SMI ranges from 6 to 18. The K6 reported reliability is α = 0.88; its sensitivity and specificity with a score of 18 and lower for diagnosing the presence of any 30-day DSM-4 disorder are 36% and 96% [34]. K6 reliability in the current sample is α = 0.82 (_for completed scale information_ = 144 of 158).

### Data analysis

Data were analyzed using SPSS version 25 (StataCorp, College Station, TX, USA). Univariate descriptive statistics and Alpha reliabilities were computed for major scales. Multiple regressions, with stepwise variable entry, were used to determine the association of personal, interpersonal, displacement and war-related factors on total-intimacy scores and the six dimensions of intimacy. The model regressed twelve potential factors, previously believed to be potentially associated with each intimacy criterion. These included: war and displacement measures, respectively—ie, PTSD symptom severity (higher scores indicating increased severity), experienced rape (yes = 1; no = 0), having escaped to Jordan with one’s spouse (yes = 1; no = 2), feeling safe in Jordan (yes = 1; no = 0), and using services provided by NGOs in Jordan (yes = 1; no = 0). Interpersonal characteristics: years of marriage, forced marriage (yes = 1; no = 0), and number of family members lived with. Personal characteristics – ie, age, gender (men = 1, women = 2), number of children, years of education, K-6 severity of serious mental illness, and economic status (very low = 1 to very high = 5). Since exposure to war events was an almost universal experience, and the PTSD-HTQ scale included symptoms endured by refugees due to the war traumatic experiences, The War Events Questionnaire (WEQ) was not included in the model as it was redundant to the PTSD-HTQ. The variables age and K-6 were entered into the models as controls in order to determine whether they altered the effects of findings potentially associated with the influence of war on intimacy. Both these variables were then excluded from the final model predicting intimacy scores and intimacy subscale scores since “age” was collinear (r = 0.89) with years of marriage (the more relevant variable in considering family dynamics) and the K-6 was highly correlated (r = -0.33) with having “experienced rape”—the more relevant variable in terms of intimacy. The consequences of having excluded these variable in the final model are discussed in the results section.

## RESULTS

### Demographics, socio-economic and displacement circumstances

The sample included 158 individual Syrian refugees who were married, 43.7% men and 56.3% women ranging in age from 19-62 (M ± standard deviation = 37.09 ± 10.03). They were married between four months to 42 years (M = 15.47 ± 10.22). Their age at marriage ranged from 13 to 36 years (M = 21.67 ± 4.68). Participants had 0-15 children (M = 4.29 ± 3.02) and 0-24 years (M = 10.26 ± 4.58) of education. Before displacement they had resided in Dara’a (34.8%), Homs (23.4%), Damascus (17.1%), Damascus countryside (7%), and other locations (17.7%). Many refugees (82.3%) escaped to Jordan with their children, and their spouses (70.9%); others made the journey with extended family members (46.2%). Before residing in Jordanian host communities, 41.4% lived in a refugee camp, most (90.6%) in the Za’atari camp located in Northern Jordan. After being displaced, they lived in Jordan between 1 day to 36 months (M = 14.19 ± 7.65) and lived with 1-15 (M = 6.26 ± 2.88) family members in the same household. Only 7.6% reported working full-time, 13.3% part-time, and 6.3% volunteered; the majority (72.8%) were unemployed, and reported that their economic status was low (30.9%) or very low (53.7%) and that the income for most (89.7%) was insufficient for living.

### War exposure, mental health and interpersonal factors potentially associated with intimacy

Most refugees (94.2%) experienced war events, such as shelling, violent acts, gunfire, damage of property, and diverse interpersonal war-related experiences. Men (97.1%) reported slightly more war-exposure than women (92%). War-related experiences included: forced separation from family members, 77.8% (82% women; 72.5% men); serious physical injury from combat situation, 18.4% (23.2% men; 14.6% women); torture, 15.8% (26.1% men; 7.9% women); disappearance or kidnapping of a spouse, 15.5% (26.1% women; 1.5% men); and the disappearance or kidnapping of a child, 11% (13.5% women; 7.6% men).

Interpersonal experiences included: forced marriage, 9.5% (13.5% women; 4.3% men); and rape due to the war, 6.4% (10.2% women; 1.4% men).

On the HTQ scale, 43% screened positive on PTSD (47.2% women; 37.3% men). HTQ scores ranged from 5 to 62 with M = 37.63 ± 10.40 (M = 36.58 ± 9.12 for women; M = 38.41 ± 11.25 for men). K-6 scores ranged from 5-25 with M = 14.99 ± 4.30 (M = 14.95 ± 4.22 for women; M = 15.03 ± 4.42 for men).

### Intimacy

Overall intimacy scores were low, ie, on average refugees neither agreed nor disagreed with positive statements related to their intimate relationships, scoring M = 2.4 ± 1.1 of a possible five. Intimacy scores were lower for refugees who screened positive on the PTSD-HTQ (M = 1.95 ± 65) compared to the ones screening negative (M = 2.23 ± 66). **Table 1** presents the association of personal, interpersonal, displacement and war-related factors with total-intimacy scores and with the six intimacy dimensions. Factors showing an effect strong enough to characterize one’s overall intimacy all reported negative relationships. In order of importance they were “forced marriage” (β = -0.32, *P* < 0.001), gender (β = -0.29, *P* < 0.001) (indicating females had lower scores), PTSD (β = -0.23, *P* = 0.002), and “number of children” (β = -0.20, *P* = 0.006). Inserting age and K-6 variables into the model describing overall intimacy did not change the results and both variables were not significant. Other factors had effects unique in association with a given intimacy dimension. Reduced sexual intimacy was associated with experienced rape (β = -0.15, *P* = 0.050); increased intellectual intimacy was associated with both increased years of education (β = 0.23, *P* = 0.003) and having escaped to Jordan with one’s spouse (β = -0.16, *P* = 0.040); increased recreational intimacy was associated with more education (β = 0.17, *P* = 0.032) and reduced in association with increased number of family members residing with one’s household (β = -0.16, *P* = 0.044). When age and K-6 were inserted into the models, the subscales of intimacy dimensions did not alter the relationship of PTSD to these intimacy dimensions. However, only increasing age was associated with increasing sexual intimacy (β = 0.59, *P* < 0.001) in this expanded model, but was not significant in any of the other intimacy dimensions. Additionally, K-6 was not significant in any of the expanded intimacy dimension models.

**Table 1:** Stepwise multiple regressions of the factors associated with married Syrian refugee individuals’ total intimacy score and its six dimensions (N = 141)

Predictor Variables	Total intimacy Score			Emotional intimacy			Social intimacy			Sexual intimacy			Intellectual intimacy			Recreational intimacy			Anger intimacy		
Beta	T	Sig.	Beta	t	Sig.	Beta	T	Sig.	Beta	t	Sig.	Beta	t	Sig.	Beta	t	Sig.	Beta	t	Sig.
PTSD-HTQ*	-0.23	-3.23	0.002	-0.17	-2.34	0.020	‡	‡	‡	-0.20	-2.68	0.008	-0.24	-3.19	0.002	-0.21	-2.76	0.007	‡	†	†
Using services provided by NGOs in Jordan	†	†	†	†	†	†	†	†	†	†	†	†	†	†	†	†	†	†	†	†	†
Feeling safe in Jordan	†	†	†	†	†	†	†	†	†	†	†	†	†	†	†	†	†	†	†	†	†
Gender	-0.29	-4.09	0.000	-0.27	-3.58	0.000	-0.24	-2.98	0.003	†	†	†	†	†	†	†	†	†	-0.49	-6.68	0.000
Years of marriage	†	†	†	†	†	†	†	†	†	†	†	†	†	†	†	†	†	†	†	†	†
Number of children	-0.20	-2.81	0.006	-0.15	-2.07	0.040	†	†	†	-0.36	-5.13	0.000	†	†	†	†	†	†	†	†	†
Number of family members lived with	†	†	†	†	†	†	†	†	†	†	†	†	†	†	†	-0.16	-2.04	0.044	†	†	†
Escaped to Jordan with spouse	†	†	†	†	†	†	†	†	†	†	†	†	-0.16	-2.03	0.04	†	†	†	†	†	†
Years of education	†	†	†	†	†	†	†	†	†	†	†	†	0.23	2.99	0.003	0.17	2.16	0.032	†	†	†
Economic status	†	†	†	†	†	†	†	†	†	†	†	†	†	†	†	†	†	†	†	†	†
Forced marriage	-0.32	-4.55	0.000	-0.31	-4.18	0.000	†	†	†	-0.27	-3.81	0.000	-0.26	-3.41	0.001	-0.25	-3.26	0.001	†	†	†
Experienced rape	†	†	†	†	†	†	†	†	†	-0.15	-1.98	0.050	†	†	†	†	†	†	†	†	†
Model statistics‡	R = 0.59; Adj.R^2^ = 0.32; df = 4137; F = 17.99; *P* < 0.001			R = 0.52; Adj.R^2 ^= .25; df = 4137; F = 12.84; *P* < 0.001			R = 0.24; Adj.R^2^ = 0.05; df = 1140; F = 8.90; *P* = 0.003			R = 0.57; Adj.R^2 ^= .31; df = 4137; F = 16.76, *P* < 0.001			R = 0.49; Adj.R^2^ = 0.22; df = 4137; F = 10.92; *P* < 0.001			R = 0.47; Adj.R^2^ = 0.19; df = 4137; F = 9.48; *P* < 0.001			R = 0.49, Adj.R^2^ = 0.24; df = 1140; F = 44.58; *P* < 0.001		

NGOs – non-governmental organizations

*PTSD-HTQ, The Harvard Trauma Questionnaire (HTQ) assesses PTSD symptoms associated with traumatic experiences.

†Order of entry and relative importance indicated by size of the beta coefficient.

‡Excluded variable.

## DISCUSSION

This study found that 43% of Syrian refugees who live in Jordanian host communities suffer from PTSD (47.2% women; 37.3% men). Personal, interpersonal, displacement and war-related factors were associated with total-intimacy scores, as well as with each of the six intimacy dimensions. PTSD and forced marriage were the strongest factors in association with decreased intimacy scores, whereas gender was the second strongest factor, meaning that women reported suffering more than men from deteriorated intimacy in their marital relationships. Higher prevalence of women screening positive on PTSD is concordant with other studies finding that women appear to be at higher risk of developing PTSD after being exposed to a traumatic event, compared to men [15,22,23].

Intimacy is identified as the sense of closeness and connection that develops through communication in couples’ interactions [1]. In this study, PTSD was associated with decreased total intimacy scores, as well as decreased emotional, sexual, intellectual and recreational intimacy. Some of the cluster-symptoms of PTSD include a sense of numbness, and avoidance of traumatic reminders [35]. It seems that suffering from PTSD may be inhibiting refugees from communicating, expressing themselves and sharing their experiences (emotional intimacy) [20], in addition to sharing their thoughts and beliefs with their spouses (intellectual intimacy). PTSD also had an impact on refugees’ sexual and recreational activities. Complex PTSD (CPTSD) includes the three cluster-symptoms for PTSD, in addition to three other clusters of disturbances in self-regulation, which includes disturbances in relationships. These disturbances are associated with prolonged and sustained forms of traumatic experiences that reflect the loss of emotional, psychological and social resources [35]. It is possible that Syrian refugees in this study suffered from CPTSD and not only PTSD, due to the war traumatic experiences and displacement challenges, which potentially affected their intimate relationships.

Forced marriages and early/child marriages have long existed in pre-war Syria due to gender inequalities, poverty and lack of opportunities for girls, but these appear to be exacerbated with displacement challenges that included social-economic pressures in Jordan [36,37]. Though early and forced marriages are the reality for both boys and girls, reports indicate that the majority of cases involve girls [36]. In this study, 13.5% women and 4.3% men experienced forced marriages, and the age at marriage ranged from 13 to 36 years. It is unknown to the researchers whether such forced marriages occurred due to post-conflict challenges in Jordan or due to previous circumstances. Reports on forced marriages argue that they are included in the definition of gender-based violence [37], and exacerbated women’s risk of experiencing intimate partner violence [17], to the extent of being considered as equivalent to enslavement crime, especially due to their direct connection to sexual abuse/slavery, lack of consent and free will [38]. Taking these reports into account and considering forced marriage as a form of legitimate and legal violence enforced within a marriage, it is no surprise that it yielded negative correlations with most of the intimacy dimensions (emotional, sexual, intellectual and recreational), and the total-intimacy score. Intimacy thrives in equal relationships where mutuality of emotions are celebrated with partners’ free will and consent.

Feminist and status inconsistency theories argue that in family systems when members are threatened by lack of resources, change of social status that is incompatible with social norms, agitated relationships and violence may occur as a strategy to compensate for losing power and control, especially against women in immigrant families [39]. A study conducted with Syrian refugees in Jordan found that increased income was associated with well-being and posttraumatic growth [40]. Syrian refugees were not allowed to work, and only in 2016 the Jordanian government issued new regulations that granted work permits to a small portion of Syrians (30,000) [41]. Therefore, men who were the main providers in Syria have lost their power in taking care of their familial responsibilities due to being uprooted. Such loss of power and control over one’s destiny and future, as well as loss of extended family and social support, may contribute to the already agitated circumstances of displacement [16] and lead to deteriorated intimate relationships.

Ethiopian refugee women reported that working enhanced their autonomy and sense of independence, provided new social networks, in addition to the economic benefits [14]. These findings add to the understanding of the deteriorated intimacy scores of Syrian refugee women, who are mainly responsible for household chores and child rearing, and are not expected to contribute to bearing the burden of work related income, not to mention their limited ability to leave their homes without a male family member or his permission [37]. Such gender-role expectations may prevent women from looking for work opportunities [42] and depend on their spouses to provide for their families. Women may feel unentitled to complain to their spouses and may tolerate unacceptable behaviors and situations, without expressing their true feelings (decreased emotional intimacy), or even their anger (anger in relationship). Women who do not work and don’t leave the house on a regular basis, would also suffer from limited social connections, which may explain their decreased social intimacy.

Rape is a weapon of war [43] and in this study, 6.4% had been raped due to the war (10.2% women; 1.4% men). Rape serves two major aims: To terrorize the civilian population, and thus forces its people to flee their country; and to cause shame and humiliation to the targeted population [44]. “The stigmatization, betrayal and abandonment associated with having been raped greatly affect the capacity of women to raise children and participate in community life. It also affects the morale of the men who perceive their inability to protect their women as one final humiliation of the war” [44; p. 174], not to mention the psychological negative impact of rape on both men and women [15,24,45]. Rape has also been identified as creating disturbances in survivors’ sexuality [45]. This study expands the body of knowledge on the negative impact of war-related rape, in adding an evidence to a deteriorated interpersonal aspect (sexual intimacy), and not only a personal one (sexuality) to the already challenged refugees’ lives.

Refugees describe their escape to the host countries as a horror journey, both in the personal and familial aspects [46]. During the escape from the war, 70.9% of refugees were with their spouses. It could be that escaping to Jordan with one’s spouse holds a horrifying experience, which partners undertook together, and thus sharing its details with one another might unite them in having a shared unique experience. Intellectual intimacy includes the experience of sharing ideas about life and work [7,8]. It seems that escaping while being in “the same boat,” brings a space for married refugees to reflect on such horrifying experience cognitively, with partners who really understand the circumstances, since they were there, and thus increasing couples’ intellectual intimacy.

Researchers claim that displaced men are stressed to gain access to work, whereas women are overburdened with household tasks and demands that they have less time to participate in social and familial activities [42]. Syrian refugees, men and women, face many challenges after being uprooted, and are extremely involved with survival needs [40] and therefore family members who live together become the only social support system they have [16]. However, the greater the number of family members living together, the greater the obligations needed to be fulfilled, which in turn leaves less time for couples to invest in recreational activities together, or that such activities are shared with family members [27]. Therefore, refugees demonstrate decreased recreational intimacy associated with increased number of family members with whom they live.

Parenting and the presence of children in a family are sources of happiness, but they are also one of the greatest sources of pressure on couples’ relationships. The investment in children decreases parents’ dyadic interactions, spontaneity, and the degree of privacy in sexual intercourse, and decreases marital happiness, especially in the early years of child-rearing when parents are physically tired and sleep deprived [47]. The number of children adds to the burden of responsibilities refugees need to carry on their shoulders. This burden of responsibility is taking the biggest tool on refugees’ sexual intimacy. It could be that the number of children is dictating the amount of space in the housing arrangement and the level of privacy couples have. Such privacy can also limit couples capacity to communicate on a daily basis, and therefore they also suffer from decreased emotional intimacy.

Years of education, was an apparent protective factor that enhanced refugees’ intellectual and recreational intimacy. It has been reported that increased education is correlated with increased marital satisfaction [48]. It seems that refugees with low resources, cannot afford going out much or spending much, and therefore their level of education can contribute to being engaged in interesting conversations with each other (intellectual intimacy) and to the spaces where they can have such conversations, which may enhance their recreational intimacy.

### Limitations

The study design has some limitations that need to be taken into consideration. First, there was no control group from which the results could have been compared. Another study that utilized the PAIR scale with Palestinian couples [27], who were not under the extreme conditions of a violent war like the Syrian refugees in this study, yielded slightly higher scores (Palestinians; women M = 2.68, men M = 2.59, compared to Syrian refugees; women M = 1.94 ± 0.67, men M = 2.40 ± 0.55). It is unknown to the authors if the lower scores of Syrian refugees are stemming from the war experiences, the displacement challenges or other factors. Second, the data collected was of convenience sampling, especially due to the complexity of collecting data from refugees and the lack of data collected on their quality of relationships before the war. Additionally, it was impossible to collect data from couples since many have escaped without their spouses and due to the fact that many have reached the study interview by themselves. It is clear to the authors that dyadic data could have provided a more comprehensive understanding of refugee intimate relationships. Third, Cronbach's alpha of social intimacy was very low (.23). It could be that social intimacy is multidimensional and may require more items to capture its full meaning especially with refugee populations who have lost many of their friends due to the war and up-rootedness and are limited in resources, which dictate their social activities. It could also be that in the Arab society, married couples are involved in social activities that involve segregation between men and women, or activities that include familial interactions, rather than social activities solely for couples.

## CONCLUSIONS

Researchers suggest that interventions that aim at resolving family violence should not only include the individual, but also the family [12]. We further suggest that couples distress and PTSD should not be addressed in individual interventions only, but also with spouses and family members, especially with Middle Eastern populations. Psychological interventions with Middle Eastern populations are related to social stigma and may contribute to the lack of service usage, despite the need for assistance [37]. We recommend that agencies working with women, encourage their arrival to the facilities with their spouses and children, by creating activities that are children-friendly, with the omission of any mental-health related titles for such activities, to minimize the pressure and social stigma related to their participation. We further recommend that interventions with refugee populations in the Middle East and elsewhere to be tailored according to their social and cultural context, in addition to being trauma-focused, and not only general therapeutic methods. Methods such as Prolonged Exposure Therapy, Cognitive Behavioral Therapy, and Dialectical Behavior Therapy are estimated to yield improvement in the mental health of populations enduring trauma and PTSD [49,50].

Additionally, we recommend raising the awareness of the negative impact of gender-based violence (rape, sexual/physical violence, early/child/forced marriages) via educating parents, caregivers, community and religious leaders, courts and judicial staff [36], in addition to staff members working in humanitarian organizations in Jordan. Since gender-based violence is still a sensitive issue, building the trust and reaching out to the community are necessary for refugees to feel comfortable to access services [37]. Moreover, creating a working model between community stakeholders and humanitarian organizations will enable faster and more trusted referrals of Syrian refugees to needed services, especially with cases of gender-based violence.

## References

[1] Laurenceau JP Barrett LF Rovine MJ The interpersonal process model of intimacy in marriage: A daily-diary and multilevel modeling approach. J Fam Psychol. 2005;19:314-23. 10.1037/0893-3200.19.2.314 15982109

[2] Calhoun PS Beckham JC Bosworth HB Caregiver burden and psychological distress in partners of veterans with chronic posttraumatic stress disorder. J Trauma Stress. 2002;15:205-12. 10.1023/A:1015251210928 12092912

[3] Dekel R Solomon Z Secondary traumatization among wives of Israeli POWs: The role of POWs’ distress. Soc Psychiatry Psychiatr Epidemiol. 2006;41:27-33. 10.1007/s00127-005-0002-6 16341620

[4] Allen ES Rhoads GK Stanlry SM Markman HJ Hitting home: Relationships between recent deployment, post traumatic stress symptoms and marital functioning for army couples. J Fam Psychol. 2010;24:280-8. 10.1037/a0019405 20545401 PMC2946188

[5] Melvin KC Gross D Hayat MJ Jennings BM Campbell JC Couple functioning and posttraumatic stress symptoms in US Army couples: The role of resilience. Res Nurs Health. 2012;35:164-77. 10.1002/nur.21459 22161808

[6] Henry SB Smith DB Archuleta KL Sanders-Hahs E Goff BS Reisbig MJ Trauma and couples: Mechanisms in dyadic functioning. J Marital Fam Ther. 2011;37:319-32. 10.1111/j.1752-0606.2010.00203.x 21745234

[7] Bagarozzi DA. Enhancing Intimacy in Marriage: A Clinician’s Guide. New York: Brunner-Routledge; 2001.

[8] Schaefer MT Olson DH Assessing intimacy: The PAIR inventory. J Marital Fam Ther. 1981;7:47-60. 10.1111/j.1752-0606.1981.tb01351.x

[9] Cigrang JA Talcott GW Tatum J Baker M Cassidy D Sonnek S Intimate partner communication from the war zone: A prospective study of relationship functioning, communication frequency, and combat effectiveness. J Marital Fam Ther. 2014;40:332-43. 10.1111/jmft.12043 24111535

[10] Gerlock AA Grimesey J Sayre G Military-related posttraumatic stress disorder and intimate relationship behaviors: A developing dyadic relationship model. J Marital Fam Ther. 2014;40:344-56. 10.1111/jmft.12017 24749950

[11] Manguno-Mire G Sautter F Lyon J Myers L Perry D Sherman M Psychological distress and burden among female partners of combat veterans with PTSD. J Nerv Ment Dis. 2007;195:144-51. 10.1097/01.nmd.0000254755.53549.69 17299302

[12] Timshel I Montgomery E Dalgaard NT A systematic review of risk and protective factors associated with family related violence in refugee families. Child Abuse Negl. 2017;70:315-30. 10.1016/j.chiabu.2017.06.023 28683372

[13] Khawaja NG Milner K Acculturation stress in South Sudanese refugees: Impact on marital relationships. Int J Intercult Relat. 2012;36:624-36. 10.1016/j.ijintrel.2012.03.007

[14] Hyman I Guruge S Mason R The impact of migration on marital relationships: A study of Ethiopian immigrants in Toronto. J Comp Fam Stud. 2008;39:149-63.

[15] Sarabwe E Richters A Vysma M Marital conflict in the aftermath of genocide in Rwanda: An explorative study within the context of community based sociotherapy. Intervention (Amstelveen). 2018;16:14-21. 10.1097/WTF.0000000000000147

[16] Betancourt TS Abdi S Ito BS Lilienthal GM Agalab N Ellis H We left one war and came to another: Resource loss, acculturative stress, and caregiver–child relationships in Somali refugee families. Cultur Divers Ethnic Minor Psychol. 2015;21:114-25. 10.1037/a0037538 25090142 PMC4315611

[17] Wachter K Horn R Friis E Falb K Ward L Apio C Drivers of intimate partner violence against women in three refugee camps. Violence Against Women. 2018;24:286-306. 10.1177/1077801216689163 29332516

[18] Schlecht J Rowley E Babirye J Early relationships and marriage in conflict and post-conflict settings: Vulnerability of youth in Uganda. Reprod Health Matters. 2013;21:234-42. 10.1016/S0968-8080(13)41710-X 23684206

[19] Charles L Denman K Syrian and Palestinian Syrian refugees in Lebanon: The plight of women and children. J Int Womens Stud. 2013;14:96-111.

[20] Shirpak KR Maticka-Tyndale E Chinichian M Post migration changes in Iranian immigrants’ couple relationships in Canada. J Comp Fam Stud. 2011;42:751-70. 10.3138/jcfs.42.6.751

[21] Jayawickreme N Mootoo C Fountain C Rasmussen A Jayawickreme E Bertuccio RF Post-conflict struggles as networks of problems: A network analysis of trauma, daily stressors and psychological distress among Sri Lankan war survivors. Soc Sci Med. 2017;190:119-32. 10.1016/j.socscimed.2017.08.027 28858697 PMC5607106

[22] Street AE Dardis CM Using a social construction of gender lens to understand gender differences in posttraumatic stress disorder. Clin Psychol Rev. 2018;66:97-105. 10.1016/j.cpr.2018.03.001 29580673

[23] Kessler RC Petukhova M Sampson NA Zaslavsky MM Wittchen H Twelve-month and lifetime prevalence and lifetime morbid risk of anxiety and mood disorders in the United States. Int J Methods Psychiatr Res. 2012;21:169-84. 10.1002/mpr.1359 22865617 PMC4005415

[24] Kessler RC Sonnega A Bromet E Hughes M Nelson CB Posttraumatic stress disorder in the national comorbidity survey. Arch Gen Psychiatry. 1995;52:1048-60. 10.1001/archpsyc.1995.03950240066012 7492257

[25] Al Ibraheem B Kira IA Aljakoub J Al Ibraheem A The health effect of the Syrian conflict on IDPs and refugees. Peace Conflict. 2017;23:140-52. 10.1037/pac0000247

[26] Hershaleg A. Intimacy and Mutual Familiarity of Emotions among Married Couples in the City and Kibbutz (Master’s thesis). Haifa University, Haifa, Israel; 1984.

[27] Rizkalla N Rahav G Differentiation of the self, couples’ intimacy and marital satisfaction: A similar model for Palestinian and Jewish married couples in Israel. Int J Juris Fam. 2016;7:1-32.

[28] Rizkalla N Zeevi-Barkai M Segal SP Rape crisis counseling: Trauma contagion and supervision. J Interpers Violence. 2017;1: 886260517736877. 10.1177/0886260517736877 29294964

[29] Karam EG Al-Atrash R Saliba S Melhem N Howard D The War Events Questionnaire. Soc Psychiatry Psychiatr Epidemiol. 1999;34:265-74. 10.1007/s001270050143 10396169

[30] Mollica RF Caspi-Yavin Y Bollini P Truong T Tor S Lavelle J The Harvard Trauma Questionnaire: Validating a cross-cultural instrument for measuring torture, trauma, and posttraumatic stress disorder in Indochinese refugees. J Nerv Ment Dis. 1992;180:111-6. 10.1097/00005053-199202000-00008 1737972

[31] Shoeb M Weinstein H Mollica R The Harvard Trauma Questionnaire: Adapting a cross-cultural instrument for measuring torture, trauma and posttraumatic stress disorder in Iraqi refugees. Int J Soc Psychiatry. 2007;53:447-63. 10.1177/0020764007078362 18018666

[32] Kessler RC Barker PR Colpe LJ Epstein JF Gfroerer JC Hiripi E Screening for serious mental illness in the general population. Arch Gen Psychiatry. 2003;60:184-9. 10.1001/archpsyc.60.2.184 12578436

[33] Kessler RC Green JG Gruber MJ Sampson NA Bromet E Cuitan M Screening for serious mental illness in the general population with the K6 screening scale: Results from the WHO World Mental Health (WMH) survey initiative. Int J Methods Psychiatr Res. 2010;19 suppl 1:4-22. 10.1002/mpr.310 20527002 PMC3659799

[34] Cornelius BLR Groothoff JW van der Klink JJL Brouwer S The performance of the K10, K6 and GHQ-12 to screen for present state DSM-IV disorders among disability claimants. BMC Public Health. 2013;13:128. 10.1186/1471-2458-13-128 23402478 PMC3575398

[35] Cloitre M Garvert DW Brewin CR Bryant RA Maercker A Evidence for proposed ICD-11 PTSD and complex PTSD: A latent profile analysis. Eur J Psychotraumatol. 2013;4:1-12. 10.3402/ejpt.v4i0.20706 23687563 PMC3656217

[36] Swan G Child marriage in Jordan: Breaking the cycle. Forced Migr Rev. 2018;57:43-4.

[37] United Nations Entity for Gender Equality and the Empowerment of women (UN Women). Inter-Agency assessment: gender-based violence and child protection among Syrian Refugees in Jordan, with a focus on early marriage; 2013. Available: http://jo.one.un.org/uploaded/publications_book/1458653027.pdf. Accessed: 4 January 2019.

[38] Haenen I The parameters of enslavement and the act of forced marriage. Int Crim Law Rev. 2013;13:895-915. 10.1163/15718123-01304005

[39] Yick AG Feminist theory and status inconsistency theory: Application to domestic violence in Chinese immigrant families. Violence Against Women. 2001;7:545-62. 10.1177/10778010122182596

[40] Rizkalla N Segal SP Wellbeing and growth among Syrian refugees in Jordan. J Trauma Stress. 2018;31:213-22. 10.1002/jts.22281 29604123

[41] Sarrado O, Dunmore C. New Deal on Work Permits Helps Syrian Refugees in Jordan. 2017. Available: http://www.unhcr.org/enus/news/latest/2017/10/59df254b4/new-deal-work-permits-helps-syrianrefugees-jordan.html. Accessed: 4 January 2019.

[42] Zetter R, Boano C. Gendering space for forcibly displaced women and children: Concepts, policies and guidelines. In: Forbes Martin S, Tirman J, editors. Women, Migration, and Conflict. Dordrecht: Springer; 2009.

[43] Rittner C, Roth JK, editors. Rape: Weapon of war and genocide. St. Paul, MN: Paragon House; 2012.

[44] Leaning J, Bartels S, Mowafi H. Sexual violence during war and forced migration. In: Forbes Martin S, Tirman J, editors. Women, migration, and conflict. Dordrecht: Springer; 2009.

[45] Herman JL. Trauma and Recovery: The aftermath of violence—from domestic abuse to political terror. New York: Basic Books; 1992.

[46] BenEzer G Zetter R Searching for directions: Conceptual and methodological challenges in researching refugee journeys. J Refug Stud. 2015;28:297-318. 10.1093/jrs/feu022

[47] Nezhad MZ Goodarzi AM Sexuality, intimacy, and marital satisfaction in Iranian first-time parents. J Sex Marital Ther. 2011;37:77-88. 10.1080/0092623X.2011.547336 21400332

[48] Mirecki RM Chou JL Elliott M Schneider CM What factors influence marital satisfaction? Differences between first and second marriages. J Divorce & Remarriage. 2013;54:78-93. 10.1080/10502556.2012.743831

[49] Foa EB, Hembree EA, Rothbaum BO. Prolonged exposure therapy for PTSD: emotional processing of traumatic experiences (therapist guide). New York, NY: Oxford University Press; 2007.

[50] Bradley R Greene J Russ E Dutra L Westen D A multidimensional meta-analysis of psychotherapy for PTSD. Am J Psychiatry. 2005;162:214-27. 10.1176/appi.ajp.162.2.214 15677582

